# Applicability of the Leiden Convention and the Lipton Classification in Patients with a Single Coronary Artery in the Setting of Congenital Heart Disease

**DOI:** 10.3390/jcdd8080093

**Published:** 2021-08-04

**Authors:** Diana Isabel Katekaru-Tokeshi, Moisés Jiménez-Santos, Claire J. Koppel, Hubert W. Vliegen, Mariana Díaz-Zamudio, Francisco Castillo-Castellón, Monique R. M. Jongbloed, Eric Kimura-Hayama

**Affiliations:** 1 Service of Cardiology, Hospital Nacional Dos de Mayo, Lima 150101, Peru; diakatekaru@hotmail.com; 2 Departament of Radiology, Service of Computed Tomography, Instituto Nacional de Cardiologia Ignacio Chavez, Juan Badiano 1, Col. Seccion XVI, Mexico City 14080, Mexico; mjimenez@ctcardiomexico.com (M.J.-S.); diazzmariana@gmail.com (M.D.-Z.); fcastillo@ctcardiomexico.com (F.C.-C.); 3 Department of Cardiology, Leiden University Medical Center, Albinusdreef 2, Postal Zone B-04-P, 2300 RC Leiden, The Netherlands; C.J.Koppel@lumc.nl (C.J.K.); h.w.vliegen@lumc.nl (H.W.V.); M.R.M.Jongbloed@lumc.nl (M.R.M.J.); 4 Department of Anatomy and Embryology, Leiden University Medical Center, Einthovenweg 20, Postal Zone S-1-P, 2300 RC Leiden, The Netherlands

**Keywords:** single coronary artery, Leiden Convention coronary coding system, Lipton classification, coronary artery anatomy, congenital heart disease

## Abstract

In single coronary artery (SCA) anatomy, all coronary tributaries arise from a single ostium, providing perfusion to the entire myocardium. Coronary classification systems can facilitate the description of SCA anatomy. **Aim:** Evaluation of the applicability of Lipton classification and the Leiden Convention coronary coding system in SCA. **Methods:** All patients (n = 6209) who underwent computed tomography (CT) scanning between 2014 and 2018 were retrospectively examined for the presence of SCA and classified, according to Lipton classification and the Leiden Convention coronary coding system. **Results:** The prevalence of SCA was 0.51% (32/6209). Twenty-eight patients (87.5%) had coexisting congenital heart disease (CHD), most frequently pulmonary atresia (9/32, 28.1%). Ten patients (10/32, 31.25%) could not be classified with either the Leiden Convention or Lipton classification (pulmonary atresia n = 9, common arterial trunk (CAT) n = 1). In one case with CAT, Lipton classification, but not the Leiden Convention, could be applied. In two cases with the transposition of the great arteries and in two cases of double outlet right ventricle, the Leiden Convention, but not the Lipton classification, could be applied. **Conclusions:** Both classifications are useful to detail information about SCA. As Lipton classification was not developed for structural heart disease cases, in complex CHD with abnormal position of the great arteries, the Leiden Convention is better applicable. The use of both systems is limited in pulmonary atresia. In this scenario, it is better to provide a precise description of the coronary origin and associated characteristics that might affect treatment and prognosis.

## 1. Introduction

In single coronary artery (SCA) anatomy, all coronary tributaries arise from a single ostium, providing perfusion to the entire myocardium [1,2]. The prevalence reported in the general population is 0.024% to 0.066%, diagnosed by invasive coronary angiography [1]. SCA has been associated with other congenital heart disease (CHD) in 40% of cases [2].

As variations in origin and course are common, classification systems have been developed to facilitate anatomical description.

**Figure 1:**
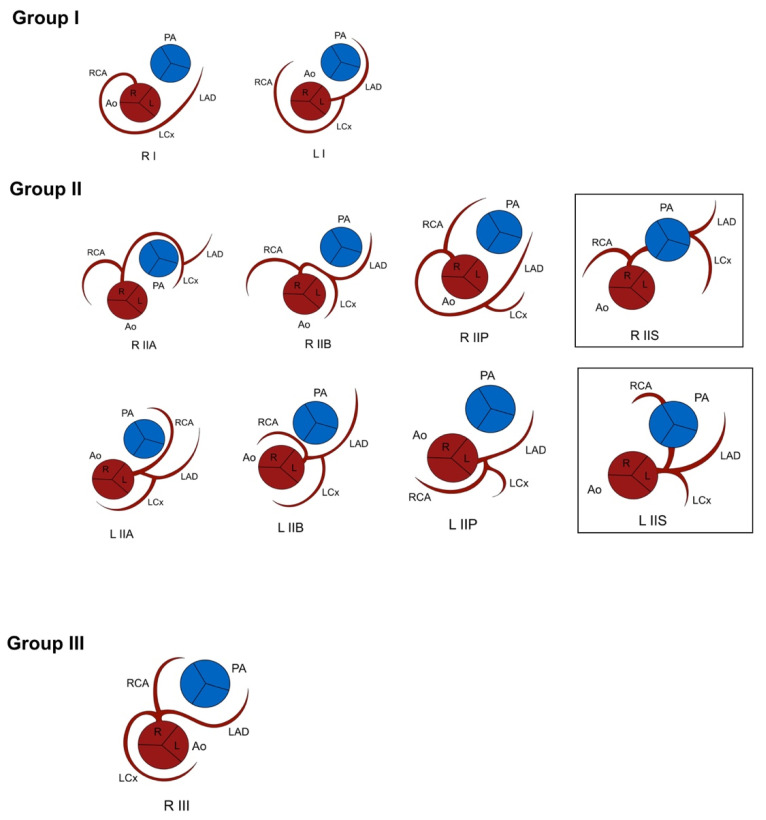
Classification of SCA by Lipton and modified by Yamanaka and Hobbs (marked). Viewed from caudal to cranial, “imaging view”. Ao: Aorta. L: Left sinus of Valsalva. LAD: Left anterior descending artery. LCx: Left circumflex artery. PA: Pulmonary artery. R: Right sinus of Valsalva. RCA: Right coronary artery.

In 1979, Lipton described a SCA classification system based on the origin, branching pattern, and course of the coronary artery [2]. The classification system describes coronary anatomy according to predefined patterns. In 1990, the system was modified by Yamanaka and Hobbs to include a septal course [3] (Figure 1). SCAs originating from the anatomical right or left sinus are indicated by an R and L, respectively, followed by the numerical category of the branching pattern (I–III). In Lipton classification, an origin of the coronary artery from the posterior sinus is not included. The classification was not primarily developed, however, for CHD with abnormally aligned great vessels.

**Figure 2:**
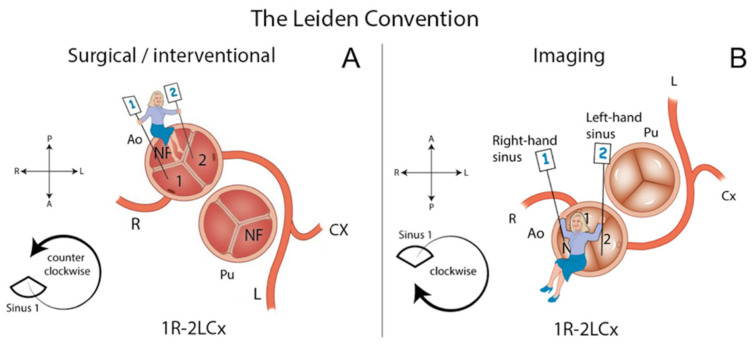
The Leiden Convention considers a surgical/interventional or an imaging perspective (Figure derived with permission from Koppel et al. [6]). (**A**) Surgical/Interventional Leiden Convention. In this method, the coronary anatomy is examined from above, as a surgeon would see it during surgery. The physician sits in the non-facing sinus, facing the pulmonary valve. In that position, the sinus on the right is sinus 1 and the sinus on the left is sinus 2. Starting from sinus 1, the coronary branches are named in the order that they are encountered when following a *counterclockwise* rotation. (**B**) Imaging Leiden Convention. The physician’s view on the coronary anatomy is from the base of the aorta upward. The physician sits in the non-facing sinus of the aortic valve, facing outward from the sinus. From this position, the right-hand sinus is again sinus 1, and the left-hand sinus is sinus 2. Following a *clockwise rotation*, starting at sinus 1, the encountered coronary branches are annotated. A: Anterior. Ao: Aorta. L: Left. NF: Non-facing sinus. P: Posterior. Pu: Pulmonary. R: Right.

The imaging Leiden Convention coronary is a coronary coding system that was developed in the 1980s by Prof. Dr. Adriana Gittenberger-de Groot [4,5]. It can be used in cases with variable and complicated coronary patterns and has the advantage that it can be used irrespective of the position of the great arteries. The system approaches the coronary anatomy based on the fact that both great arteries have, in principle, two “facing” sinuses (Figure 2). Therefore, it applies in structurally normal hearts as well as in CHD, including bicuspid aortic valves. The system avoids using terms related to the spatial orientation of the arterial origin from the aorta in the description, and designates the aortic sinuses as “left or right facing” or “non-facing” relative to the pulmonary valve sinuses. This is based on the principle that the non-facing sinus (corresponding to the posterior sinus in the case of a normal position of the great arteries), will never carry a coronary artery. Originally, the system was developed for use by cardiac surgeons but was recently extended to provide its use by interventional and imaging (pediatric) cardiologists as well [5,6]. To correct for the different angles at which the coronary arteries are viewed by surgeons and imaging cardiologists, the methods to acquire the same annotation are slightly different for the imaging than for the surgical perspective of the Leiden Convention coding system. For the imaging view, the physician positions themselves in the non-facing sinus of the aortic valve, looking out from the sinus. Positioned in this way, the right-hand sinus is sinus 1, and the left-hand sinus is sinus 2. Then, the branches are described in the order in which they are encountered when adopting a clockwise rotation starting at sinus 1 [5,6] (Figure 2). To our knowledge, there are no studies that evaluate the applicability of the two classification systems (i.e., Lipton classification and the Leiden Convention coronary coding system) for classification of single coronary arteries in the setting of complex congenital heart disease. The aims of the present study are (1) to report the prevalence of cases of SCA diagnosed by coronary computed tomography angiography (CCTA) in a tertiary healthcare cardiovascular center and (2) to evaluate the applicability of Lipton classification and the imaging Leiden Convention coronary coding system to annotate the SCA anatomy.

## 2. Materials and Methods

Consecutive patients that were scanned between March 2014 and January 2018 in a tertiary referral center were evaluated for the presence of SCA. We employed a dual-source 256-MDCT scanner (Definition Flash, Siemens Healthineers, Erlangen, Germany) with a sectional collimation of 2 × 128 × 0.6. Either ECG-gated or non–ECG-gated high-pitch protocols were used (pitch factor of 0.17 and 3.4 respectively), at the discretion of the attending physician, taking into account the patient’s ability to follow instructions, clinical condition and request. The indication for the CCTA in pediatric patients (24/32, 75%) was to further investigate previously diagnosed CHD. The indication for the CCTA in the adult patients (8/32, 25%) was to rule out coronary artery disease. In cardiac-gated scans, a prospective acquisition was triggered in systole (40–45% of R-R interval). X-ray tube parameters of voltage and current were adjusted to patient’s weight as follows: 80 kV if less than 20 kg and 100 kV if BMI < 25 or weight 20–80 kg, with a current of 10 mA/kg (up to 9 kg) and 5 mA for each additional kg. A current *z-*modulation technique (CARE-Dose, Siemens Healthinneers) was used in all patients. The FOV extended from the carina to the lower portion of the heart. The contrast transit time was determined with the use of a bolus tracking technique by placing the region of interest in the ascending aorta. Images were reconstructed in 0.75 mm thickness and interval reconstruction with a B25f kernel filter.

We used non-ionic contrast material (iobitridol, Guerbet, Roissy, France) at a volume of 1–1.5 mL/kg of body weight for non-gated scans or 1.5–2 mL/kg for ECG-gated acquisitions. All images were analyzed by an imaging cardiologist with 8 years of experience in adult and pediatric cardiovascular imaging (MJS). Afterwards, the coronary anatomy was also reviewed and annotated by CJK. Dedicated cardiac workstations were used (Syngo Via v2.0, Siemens Healthineers, Forcheim, Germany and Vitrea, Vital images, Inc., Minnetonka, MN, USA., resp.), capable of multiplanar and volume rendering reformations. An institutional review board (IRB) consent form was obtained at the time that CCTA was performed. The average dose length product (DLP) was 142.5 mGy-cm (16–426 mGy-cm).

## 3. Results

The prevalence of SCA diagnosed by CCTA was 0.51% (32/6209) in this tertiary cardiovascular referral center. Twenty patients were female (62.5%); the median age was 16 years and 7 months (range 1 month to 78 years) (Table 1).

**Figure 3:**
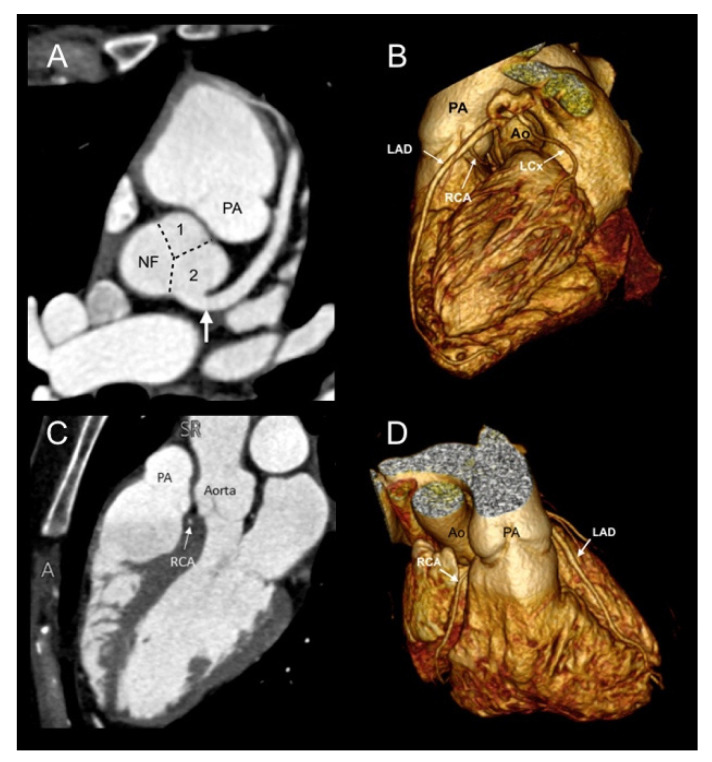
Case 27. CCTA in a 37-year-old woman undergoing preoperative evaluation for an atrial septal defect. (**A**) Axial image depicting the origin of the SCA (arrow) from sinus 2. (**B**) A 3D volume-rendered image showing that the RCA originates from the middle segment of the LAD with an interarterial course. According to the Imaging Leiden Convention, the anatomy is 2R*LCx; by Lipton classification, it is LIIB. (**C**,**D**) The RCA follows a course between the aorta and pulmonary artery (level of right ventricular outflow tract). Ao: Aorta. LAD: Left anterior descending coronary artery. LCx: Left circumflex. NF: Non-facing sinus. PA: Pulmonary artery. RCA: Right coronary artery.

Ten patients (31.25%) presented with a concomitant coronary anatomy anomaly (CAA), including an anomalous origin of a right coronary artery (RCA) from the left anterior descending coronary artery (LAD) (Figure 3) (6/32, 18.75%) and an absent RCA (4/32, 12.5%). Of the 32 included patients, 4 (12.5%) showed an interarterial course.

Twenty-eight patients (87.5%) had coexisting CHD (Table 1). The most frequent were pulmonary atresia/agenesis (9/32, 28.1%), one of which also had a double-outlet right ventricle, right aortic arch (6/32, 18.75%), double-outlet right ventricle (4/32, 12.5%), tetralogy of Fallot (4/32, 12.5%), and transposition of great arteries (3/34, 9.4%).

**Figure 4:**
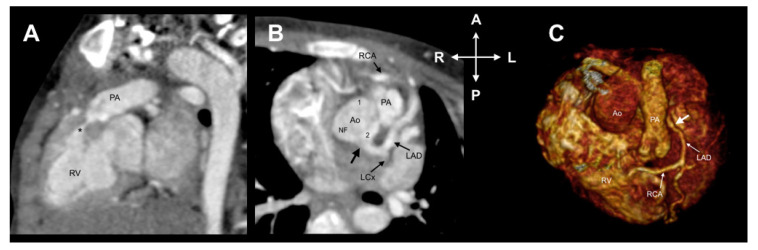
Case 9. A 4-year-old child with tetralogy of Fallot. (**A**) Oblique image showing infundibular pulmonary stenosis (*). (**B**) Axial image reveals coronary origins clockwise rotated along with the aortic root. According to the Leiden Convention, it is 2RLCx and by Lipton classification, it is LIIA. (**C**) A 3D volume-rendered image shows an anomalous origin of the RCA from the LAD. A: Anterior. Ao: Aorta. LAD: Left anterior descending coronary artery. L: Left. LCx: Left circumflex. P: Posterior. R: Right. RV: Right ventricle. NF: Non-facing sinus. PA: Pulmonary artery. RCA: Right coronary artery.

In our cohort, six patients (18.75%) had an anomalous position of the great arteries in which the anatomical relationship of the aorta in relation to the pulmonary trunk was as follows: right anterior 3 (9.4%) and left anterior 3 (9.4%). Additionally, four patients with tetralogy of Fallot had clockwise rotation of the aortic root (Figure 4).

Of the adult patients, (8/32, 25%) the mean calcium score was 11.38 Agatston units (AU) (0–63.4 AU).

### 3.1. Lipton Classification

**Figure 5:**
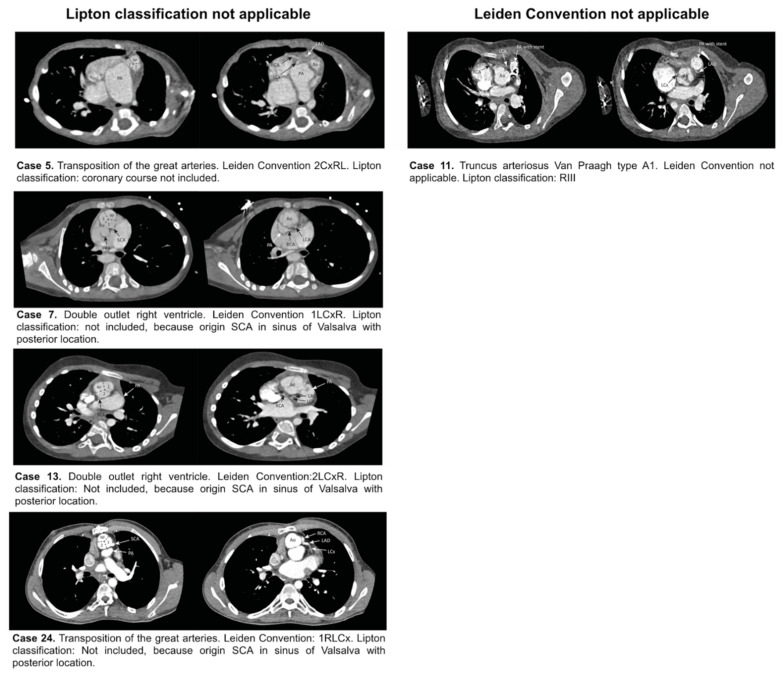
Overview of cases that could be classified only with the Lipton classification or the Leiden Convention. Ao: Aorta. LAD: Left anterior descending coronary artery. LCx: Left circumflex. R: Right. NF: Non-facing sinus. PA: Pulmonary artery. RCA: Right coronary artery. SCA: Single coronary artery.

The most common origin of the SCA was from the left anterior sinus of Valsalva (13/32, 50%), followed by an origin from the right anterior sinus of Valsalva (5/32, 15.6%), as observed from the anatomical position of the heart in the thorax. According to this system, these 18 patients were classified as LI (*n* = 4, 22.2%), LIIA (*n* = 6, 33.3%), LIIB (*n* = 2, 11%), LIIP (*n* = 1, 5.5%), RIIA (*n* = 1, 5.5%), RIIP (*n* = 1, 5.5%), RIIS (*n* = 1, 5.5%) and RIII (n = 2, 11%). However, 9 patients with CHD and abnormally aligned great vessels had a SCA origin from the anatomically posterior sinus and 4 with pulmonary atresia, and 1 patient with a common arterial trunk Van Praagh type A4 did not have a course around the pulmonary artery or right ventricular outflow tract, so could not be included in any category of the Lipton classification (Figure 5, Table 2).

### 3.2. Leiden Convention Coronary Coding System

According to the imaging Leiden Convention, the origin of the SCA was from the left-hand sinus (sinus 2) in 15 patients (71.4%). In 13 patients, these single coronary arteries were also designated as originating from the left sinus in the Lipton classification; 2 could not be classified with Lipton. The origin of the SCA was from the right-hand sinus (sinus 1) in 6 cases (28.5%), according to the Leiden Convention. This corresponded to the right sinus in the Lipton classification in 4 of these patients; 2 could not be classified according to Lipton. The imaging Leiden Convention could not be used in 11/32 patients (34%): nine patients had pulmonary atresia/agenesis, and 2 patients had a common arterial trunk (1 Van Praagh type A1 and 1 with Van Praagh type A4 [7]) (Table 2). As these conditions do not allow to determine facing and non-facing sinuses, the Leiden Convention coronary coding system could not be used for these cases.

**Figure 6:**
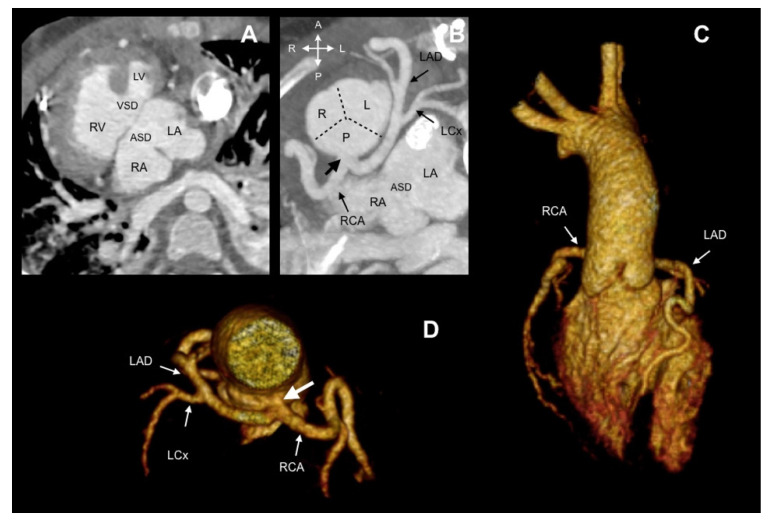
Case 17. A 10-year-old child with pulmonary atresia and common atrioventricular canal type C of Rastelli. (**A**) Oblique axial view showing atrial situs inversus, common atrioventricular connection, atrial and ventricular septal defect. (**B**) The SCA (thick arrow) arises from the posterior sinus, which gives origin to the RCA and left main stem. (**C**,**D**) A 3D volume-rendered image showing a posterior SCA. Ao: Aorta. ASD: Atrial septal defect. LA: Left atrium. LAD: Left anterior descending coronary artery. LCx. Left circumflex. LV: Left ventricle. NF: Non-facing sinus. RA: Right atrium. RCA: Right coronary artery. RV: Right ventricle. VSD: Ventricular septal defect.

In summary, 10 patients (10/32, 31.25%) could not be classified with either the imaging Leiden Convention or Lipton classification (Table 2). Nine had pulmonary atresia, 1 had a truncus arteriosus Van Praagh type 4A, and in 6 of these, the origin of the SCA was from the anatomically posterior sinus, near the interatrial septum (Figure 6). Four patients, 2 with TGA and 2 with double outlet right ventricle, could be classified by the Leiden Convention, but not by Lipton classification, and 1 patient could be classified by Lipton classification, but not by the Leiden Convention (Figure 5, Table 2). This was a patient with a truncus arteriosus Van Praagh type A1.

## 4. Discussion

We present a case series of SCA diagnosed with CCTA in a tertiary referral center. To our knowledge, this represents the largest cohort of SCA reported based on CT. The CCTA allows accurate non-invasive evaluation of the coronary ostial morphology, course, and distribution area of the coronary arteries. This might be a result of the type of institution we represent, i.e., a nationwide cardiovascular referral center for complex congenital heart disease, that may show a higher prevalence of SCA as compared to the population without structural cardiac defects. In addition, the higher prevalence of the anomaly when compared to invasive angiography might be the result of the multiplanar capabilities of CT when compared to a biplanar method with limited visualization of spatial relationships.

Coronary artery anomalies are potentially relevant findings during surgery and for coronary intervention planning. In terms of clinical diagnosis, in our series, this finding was incidental in all cases. Although none of the adult patients in this study had significant atherosclerotic disease, the detection of atherosclerosis in an SCA is important because of the potential clinical implications of significant coronary artery disease in a single vessel providing the entire myocardial blood flow. In addition, relevance of its diagnosis also lies in the fact that this anomaly may cause myocardial ischemia by different mechanisms: due to angulations or abnormal courses that may affect the distribution of blood flow, even in the absence of atherosclerotic coronary lesions [2,8,9,10].

Of the 32 patients included, only 4 (12.5%) showed an interarterial course. This variation might increase the risk of a major cardiac event, due to potential hemodynamic compromise. This prevalence is similar to previous reports [10,11]. Additionally, it is worthwhile to notice that approximately one third of the patients had an additional anomalous coronary branching pattern. In our series, associated CHD was found in almost 87.5% of cases, and concomitant anomalies in coronary arterial branching patterns were present in 31.25% of the patients.

Knowledge of anatomical variations in the origin and course of the coronary arteries in patients with CHD is essential to achieve optimal surgical preparation and follow up. Coronary classification systems are useful tools to detail important information about coronary anomalies to cardiologists, radiologists and cardiac surgeons.

Lipton classification was originally designed for patients with normal cardiac anatomy without additional congenital cardiac anomalies and is based on classifying the coronary artery as left or right, based on the spatial position (i.e., left or right) of the sinuses of Valsalva from which they emerge. To assign the branching pattern to a group, the system takes into account the coronary artery course relative to the pulmonary artery [2,3], which one might extend to the course around the right ventricular outflow tract for practical reasons. However, this limits their use in hearts with a rotated orientation of the great arteries (described as a general feature in congenital malformation of the outflow tract [12]) or pulmonary atresia. Another limitation of Lipton classification is that it contains a pre-set array of variations, leaving no room for anatomical variations outside this set. However, for many cases, the variations described by Lipton et al. are sufficient. An advantage with respect to the Leiden Convention is that posterior, anterior and septal courses of the coronary artery are directly included in the annotation and not only an interarterial course.

The Leiden Convention coronary coding system was developed to avoid using terms referring to the spatial anatomical position, such as left, right, posterior and anterior, which might be ambiguous in cases with malposition of the great arteries; herein lies its strength for use in (complex) CHD. In Lipton classification, the posterior sinus would, in many cases, correspond to the non-facing sinus in the Leiden Convention, which does not carry a coronary artery. However, especially in malrotation of the great arteries, this correspondence of the posterior sinus to the non-facing sinus is not obligatory.

The nomenclature of the sinuses of the Leiden Convention allows its use also in cases with congenital heart disease and malposition of the great arteries, regardless of the position of the great arteries and the spatial position of the sinuses of Valsalva. As the recognition of facing and non-facing sinuses is a prerequisite of the Leiden Convention coronary coding system, in principle, it cannot be used in cases of common arterial trunk, pulmonary atresia, cases with uni/quadri/pentacuspid valves or if the valve morphology cannot be distinguished [4,5]. The Leiden Convention also cannot be used if the coronary artery arises from the non-facing sinus. We have, to date, not encountered this situation in humans.

Neither the Leiden Convention nor Lipton classification could be applied in 10 patients (Table 2). Nine patients had pulmonary atresia and one patient had a common trunk Van Praagh type A4. For application of the Lipton classification, the SCA has to course around the pulmonary trunk or right ventricular outflow tract, so in the absence of a pulmonary trunk, such as in cases of pulmonary atresia, Lipton classification cannot be used. In addition, cases with the SCA originating from the posterior sinus make Lipton classification inapplicable. A coronary artery originating from a non-facing sinus is extremely rare, and we, therefore, do not consider the posterior sinus to be the equivalent of the non-facing sinus in these cases.

In some cases of pulmonary atresia where a small pulmonary trunk can be visualized, the Leiden Convention might still be applicable. However, we believe that in highly complex CHD where the coronary anatomy cannot be adequately described according to a classification system, it is better to provide a detailed description, including the origin, course (especially important in the case of the interarterial course), coronary artery dominance, the relationship relative to the position of the great vessels, as well as, in particular cases, the shape of the coronary orifice, acute angle take-off, or presence of myocardial bridging of a coronary artery. This corresponds to the “associated anomalies” step of the Leiden Convention coronary coding system.

## 5. Study Limitations

This a retrospective study including patients for whom CCTA was performed on a variety of indications. Therefore, not all scans were made in an ECG triggered way that would be optimal for evaluation of the coronary anatomy. This may have hampered an optimal evaluation in some of the cases described.

## 6. Conclusions

Both Lipton classification and the Leiden Convention are useful to present important information about coronary anomalies. In complex CHD with an abnormal position of the great arteries, the Leiden Convention is better applicable. As expected, Lipton classification, which was principally developed for cases without structural heart disease, is less suitable for complex CHD with abnormal position of the great arteries. In pulmonary atresia, the use of both systems is limited. In such cases, it is recommended to provide a detailed description of the coronary anatomy as well as associated characteristics that might affect treatment planning and prognosis.

## Figures and Tables

**Table 1:** Patients’ demographics, associated congenital heart disease, imaging Leiden Convention and Lipton classification.

Patient Number	Gender	Age	Associated Anomalies	Anatomical Relationship of the Ao to the PA	Leiden Convention	Lipton Classification	Concomitant Coronary Anomaly
1	M	1M	Double-outlet right ventricle	Right anterior	2LCx	LI	Absent RCA
2	F	2M	Truncus arteriosus Van Praagh type A4	NA	NA	NI*	None
3	F	6M	Pulmonary atresia. Single outlet right ventricle. Right aortic arch.	NA	NA	NI*	None
4	F	6M	Pulmonary atresia. Single outlet right ventricle.	NA	NA	NI*	None
5	F	8M	Transposition of the great arteries	Left anterior	2CxRL	NI*	None
6	F	3Y	Tetralogy of Fallot	Clockwise rotation of the aortic root	2RLCx	LIIA	Anomalous origin of RCA from LAD
7	M	3Y	Double-outlet right ventricle. Common atrioventricular canal type C of Rastelli. Right aortic arch	Left anterior	1LCxR	NI*	None
8	F	4Y	Stenosis left ventricular outflow tract by fibromuscular protrusion	Right posterior	2LCx	LI	Absent RCA
9	M	4Y	Tetralogy of Fallot	Clockwise rotation of the aortic root	2RLCx	LIIA	Anomalous origin of RCA from LAD
10	M	4Y	Pulmonary atresia. Ambiguous atrioventricular connection. Right aortic arch	NA	NA	NI*	None
11	F	5Y	Truncus arteriosus Van Praagh type A1. Right aortic arch	NA	NA	RIII	None
12	F	5Y	Syndrome Noonan	Right posterior	2RLCx	LIIA	None
13	F	6Y	Double-outlet right ventricle	Right anterior	2LCxR	NI*	None
14	F	8Y	Isolated	Right posterior	1RLCx	RIIS	None
15	F	9Y	Double-outlet right ventricle. Pulmonary atresia	NA	NA	NI*	None
16	M	10Y	Tetralogy of Fallot	Clockwise rotation of the aortic root	1RLCx	RIIA	None
17	F	10Y	Pulmonary agenesis. Common atrioventricular connection. Single outlet right ventricle	NA	NA	NI*	None
18	F	11Y	Pulmonary atresia	NA	NA	NI*	None
19	M	12Y	Pulmonary agenesis. Single outlet right ventricle. Right aortic arch	NA	NA	NI*	None
20	F	13Y	Mitral valve prolapse	Right posterior	1RL*Cx	RIII	None
21	M	14Y	Transposition of the great arteries	Left anterior	1LCxR	RIIP	None
22	M	15Y	Supravalvular aortic stenosis	Right posterior	2R*LCx	LIIB	None
23	M	15Y	Tetralogy of Fallot	Clockwise rotation of the aortic root	2RLCx	LIIA	Anomalous origin of RCA from LAD
24	M	16Y	Transposition of the great arteries	Right anterior	1RLCx	NI*	None
25	F	18Y	Pulmonary atresia	NA	NA	NI*	None
26	F	25Y	Pulmonary atresia. Double inlet left ventricle. Right aortic arch	NA	NA	NI*	None
27	F	37Y	Atrial septal defect. Ventricular septal defect	Right posterior	2R*LCx	LIIB	Anomalous origin of RCA from LAD
28	F	51Y	Left ventricular non-compaction	Right posterior	2RLCx	LIIA	Anomalous origin of RCA from LAD
29	M	52Y	Isolated	Right posterior	2LCx	LI	Absent RCA
30	M	53Y	Dysplastic aortic valve	Right posterior	2LCx	LI	Absent RCA
31	M	58Y	Coronary artery disease. Rupture of right sinus of Valsalva aneurysm	Right posterior	2LCxR	LIIP	None
32	F	78Y	Isolated	Right posterior	2RLCx	LIIA	Anomalous origin of RCA from LAD

Ao: Aorta. PA: Pulmonary artery. NA: Not applicable. NI*: Not included in the classification.

**Table 2:** Overview of cases not assessable by either classification.

	Lipton Classification	Leiden Convention
Patient Number	Associated Anomaly Preventing Use of Both Classification Systems	
2	Posterior sinus	Truncus arteriosus type A4
3	Posterior sinus	Pulmonary atresia
4	Course not included	Pulmonary atresia
10	Course not included	Pulmonary atresia
15	Posterior sinus	Pulmonary atresia
17	Posterior sinus	Pulmonary atresia
18	Posterior sinus	Pulmonary atresia
19	Course not included	Pulmonary atresia
5	Course not included	Pulmonary atresia
26	Posterior sinus	Pulmonary atresia
	Lipton classification applicable, Leiden Convention not applicable (Figure 5)	
11	RIII	Truncus arteriosus type A1
	Lipton classification not applicable, Leiden Convention applicable (Figure 5)	
5	Course not included	2CxRL
7	Posterior sinus	1LCxR
13	Posterior sinus	2LCxR
24	Posterior sinus	1RLCx
